# MRSA in Tibet: high prevalence driven by the epidemic clone CC59-ST59-SCC*mec* IV-t437 and insights from whole-genome analysis

**DOI:** 10.3389/fmicb.2026.1889907

**Published:** 2026-08-12

**Authors:** Wei Li, Yichuan Qiu, Xiaodong Wu, Jinmei Zhang

**Affiliations:** 1 Department of Pediatric Cardiovascular Surgery, Children’s Heart Center, West China Second University Hospital, Sichuan University, Chengdu, China; 2 Department of Clinical Laboratory, Hospital of Chengdu Office of People’s Government of Xizang Autonomous Region (Hospital.C.X.), Chengdu, China; 3 Changdu People’s Hospital of Xizang, Changdu, China

**Keywords:** CA-MRSA, HA-MRSA, molecular epidemiology, Tibetans, virulence, whole-genome sequencing

## Abstract

**Objectives:**

Methicillin-resistant *Staphylococcus aureus* (MRSA) poses a serious public health burden in Tibet, where genome-informed epidemiological data remain limited. This study aimed to characterize the molecular epidemiology of MRSA among Tibetans and explore factors associated with its elevated prevalence.

**Methods:**

We analyzed 115 non-duplicate MRSA isolates from Tibetans, comprising 51 hospital-associated MRSA (HA-MRSA) from clinical infections and 64 community-colonizing MRSA (CA-MRSA). CA-MRSA isolates were obtained from nasal swabs of hospitalized patients within 24 h of admission (to exclude nosocomial acquisition) and from their family members between June and December 2023. All isolates underwent antimicrobial susceptibility testing and whole-genome sequencing (WGS)-based typing [sequence type (ST), *spa*, *SCCmec*]. We performed core-genome single nucleotide polymorphism (cgSNP) phylogeny and profiled resistance and virulence determinants.

**Results:**

The MRSA nasal colonization rate was 10.53%. The clone CC59-ST59-SCC*mec* IV-t437 predominated, accounting for 59.4% of CA-MRSA and 39.2% of HA-MRSA. Phylogenetic analysis revealed a distinct Tibetan MRSA cluster with limited genetic relatedness to strains from other Chinese provinces. cgSNP analysis identified seven CA-MRSA transmission clusters defined by ≤25 cgSNP differences, approximating the ≤25 wgSNP threshold. a finding corroborated by epidemiological links to shared households or hospital exposure. While ST59 conserved core virulence determinants (capsule biosynthesis, iron acquisition), ST22 uniquely harbored the *egc* cluster and was uniformly positive for the *lukS-PV/lukF-PV*loci. No significant virulence differences were observed between HA-MRSA and CA-MRSA within the same ST. All isolates remained susceptible to vancomycin and linezolid, exhibiting concordant resistance profiles across both settings.

**Conclusion:**

CC59-ST59-SCC*mec* IV-t437 is the predominant MRSA lineage in Tibet and shows substantial clonal overlap between HA-MRSA and CA-MRSA. These results support the use of whole-genome sequencing to guide empirical therapy and highlight the need for continued surveillance and targeted infection control in this high-burden region.

## Introduction

Methicillin-resistant *Staphylococcus aureus* (MRSA) is a major clinical pathogen that poses a persistent and severe threat to global public health (Lee et al., 2018), being a leading cause of both hospital-associated (HA) and community-associated (CA) bacterial infections-including life-threatening conditions such as sepsis, pneumonia, and necrotizing skin and soft tissue infections (Lakhundi and Zhang, 2018). The advent of whole-genome sequencing (WGS) has transformed how MRSA transmission networks are resolved, allowing fine-scale tracking of both micro-evolution and intercontinental spread using whole-genome sequencing approaches (Harris et al., 2010). In China, while national surveillance programs, including the China Antimicrobial Resistance Surveillance System (CARSS) and CHINET, have reported a gradual decline in MRSA detection rates (from 36% in 2014 to 28.4% in 2024) (China Antimicrobial Resistance Surveillance System, 2021, 2026), *the Tibetan population in the Tibet Autonomous Region shows a very different pattern. CARSS indicate that local MRSA detection rates have remained consistently high (48.5% in 2021, 42.9% in 2023, and 47.0% in 2024), with both nasal colonization rates and clinical isolation rates markedly higher than national averages. The regional detection rate has stayed above 42.9% in recent years and consistently ranks among the top three nationwide* (China Antimicrobial Resistance Surveillance System, 2023, 2026)

This notably high MRSA burden poses major challenges for prevention and control efforts in Tibet. An important gap in addressing this issue is the lack of comprehensive, genome-scale epidemiological data-including key information on local MRSA molecular characteristics (e.g., dominant clones, antimicrobial resistance profiles), the genetic correlation between community colonization isolates (CA-MRSA) and HA-MRSA, and the evolutionary relationship of Tibetan MRSA with strains from other Chinese provinces.

To fill these gaps, this study aimed to systematically characterize the molecular epidemiological features of MRSA strains circulating among Tibetans. Specifically, we: (1) analyzed the genomic sequences of MRSA isolates from nasal colonization (CA-MRSA) and clinical infections (HA-MRSA) to clarify the genetic links between these two subtypes; (2) compared whole-genome sequences (WGS) of Tibetan MRSA with MRSA genomes from other regions of China retrieved from the NCBI database; and (3) integrated data on antimicrobial resistance, virulence genes, and phylogenetic relationships to explore factors associated with the elevated MRSA colonization and infection rates in Tibet.

## Materials and methods

### Isolation and identification

From January to December 2023, two types of MRSA isolates were collected from Tibetan individuals at the Hospital of the Chengdu Office of the People’s Government of the Tibetan Autonomous Region (Chengdu, Sichuan, China).

Hospital-associated MRSA isolates were obtained from clinical specimens (including wound secretions, sputum, and blood) of hospitalized patients collected more than 48 h after admission (to distinguish from community-acquired infections present on admission). In contrast, community colonization isolates (CA-MRSA) were collected from nasal cavity swabs of hospitalized patients within 24 h of admission (to exclude hospital-acquired colonization) and from their family members between June and December 2023. All samples were cultured on Mannitol Salt Agar (MSA; Hopebio, Qingdao, China) and incubated aerobically at 37 °C for 24–48 h. Confirmed MRSA isolates were classified as HA-MRSA or CA-MRSA according to the collection timing, in accordance with the Clinical and Laboratory Standards Institute (CLSI) M100, 33rd edition (2023) (Clinical Laboratory Standards I, 2023) and NHSN definitions (Horan et al., 2008) applicable at the time of collection and identification. Throughout this manuscript, the term CA-MRSA is used exclusively to refer to these community colonization isolates for brevity. We acknowledge that comparing colonization isolates with clinical infection isolates may introduce epidemiological bias when interpreting transmission dynamics. All suspected MRSA isolates were re-identified to the species level using Matrix-Assisted Laser Desorption/Ionization Time-of-Flight Mass Spectrometry (MALDI-TOF MS; Thermo Fisher Scientific, Cleveland, OH, United States), and cefoxitin resistance was confirmed by the disk diffusion method.

### Ethical compliance

The study protocol was approved by the Ethics Committee of the Hospital of the Chengdu Office of the People’s Government of the Tibetan Autonomous Region (Approval No. 2025032). All procedures were performed in accordance with relevant national guidelines/regulations. Written informed consent was obtained from all participants or their legal representatives (for minors or individuals with impaired decision-making capacity, if applicable).

### Antimicrobial susceptibility testing

Susceptibility to 13 antibiotics was tested for all isolates using standard disk diffusion method. *Staphylococcus aureus* ATCC 25923 was used as the quality control. The antibiotics tested included: chloramphenicol (CHL, 30 μg), gentamicin (CN, 120 μg), ciprofloxacin (CIP, 5 μg), penicillin (PEN, 10 μg), clindamycin (CLI, 2 μg), sulfamethoxazole/trimethoprim (SXT, 25 μg), teicoplanin (TEC, 30 μg), erythromycin (ERY, 15 μg), nitrofurantoin (F, 300 μg), rifampin (RIF, 5 μg), tetracycline (TET, 30 μg), linezolid (LZD, 30 μg), and cefoxitin (FOX, 30 μg). The isolates were categorized as susceptible, intermediate resistant, or resistant according to the recommendations of CLSI M100, 33rd edition (2023).

### Genome sequencing and analysis

Total DNA from the MRSA isolates was extracted using QIAamp DNA Mini Kit (Qiagen) and purified DNA was subjected to WGS using a paired-end library with an insert size of 150 bp on a NovaSeq 6000 sequencer (Illumina, San Diego, CA, United States),and WGS quality metrics are provided in Supplementary Table 1. The short Illumina reads were de novo assembled using SPAdes v4.2.0 (Bankevich et al., 2012). The assembled contigs were used for molecular typing. Specifically, sequence types (STs) were identified using the *S. aureus* multilocus sequence typing (MLST) database.^1^ The spa typing was performed using spaTyper v1.0.^2^ SCCmec typing was performed using SCCmecFinder v1.2^3^ with default settings. The presence of virulence genes was investigated by using VirulenceFinder^4^ and the virulence factor database (VFDB)^5^.

### Phylogenetic analysis

Genome sequences of *S. aureus* isolates from China were retrieved from GenBank. We assessed genetic relationships using core-genome SNPs (cgSNPs). Briefly, genomes were annotated with Prokka (Seemann, 2014), and the resulting GFF3 files were processed using Roary (Page et al., 2015) to generate a core genome alignment. We extracted cgSNPs using SNP-sites v2.3.2 (Page et al., 2016) and constructed a maximum-likelihood phylogenetic tree using FastTree v2.1.10 (Price et al., 2010). Transmission clusters were defined as groups of isolates differing by ≤25 cgSNP differences, a proxy for the widely accepted ≤ 25 wgSNP threshold for recent transmission (Coll et al., 2020). Although phylogenetic inferences were derived strictly from core-genome alignments, we applied this wgSNP threshold as a benchmark given the high concordance between core-genome and whole-genome distances in short-term outbreaks.

## Results

### Clinical characteristics of the MRSA isolates

**FIGURE 1:**
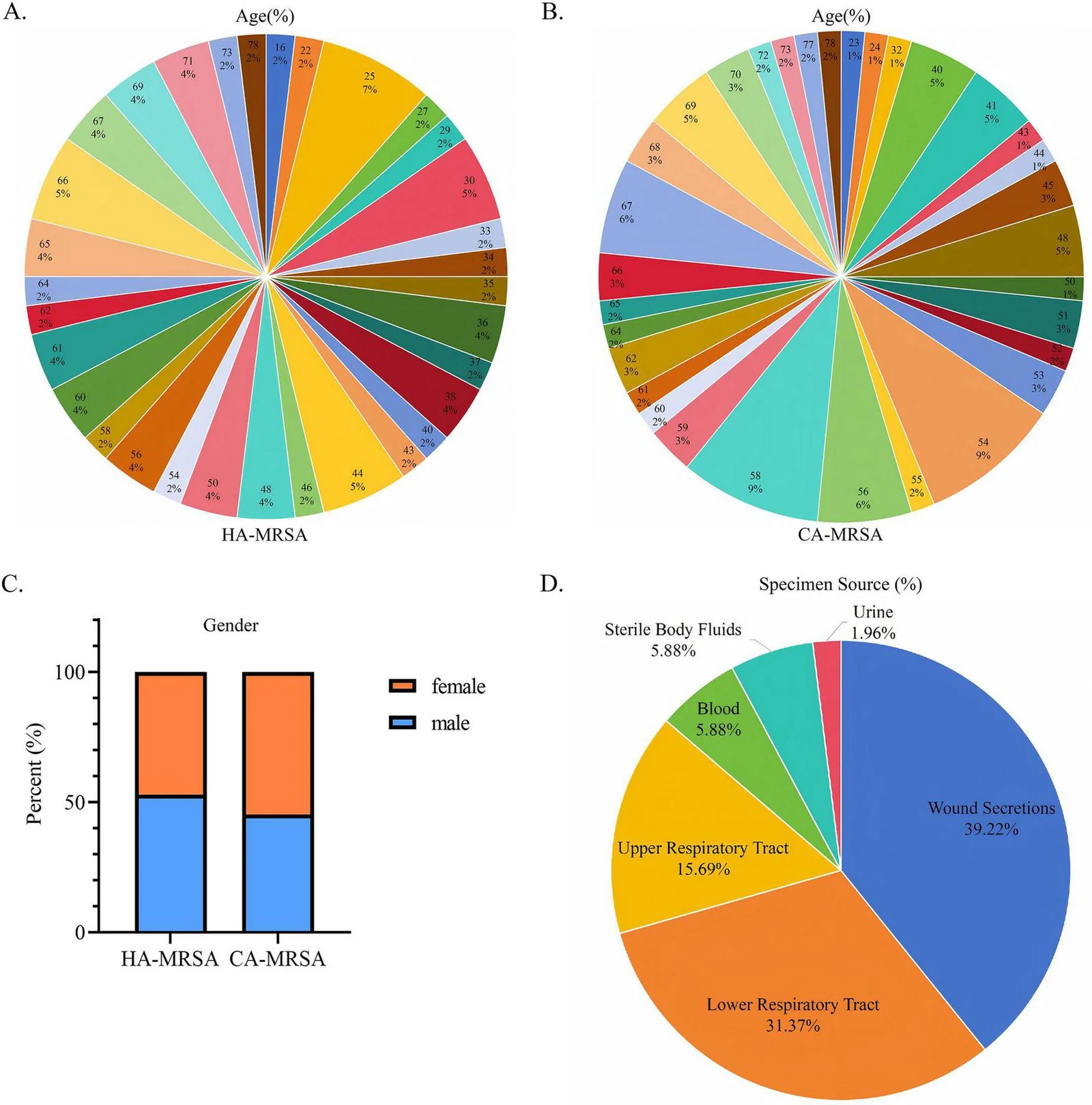
Information on sample collection and clinically isolated strains. **(A)** Age distribution and proportions of hospital-associated methicillin-resistant *Staphylococcus* aureus (HA-MRSA)-infected patients. **(B)** Age distribution and proportions of community-colonizing methicillin-resistant *Staphylococcus aureus* (CA-MRSA) carriers. **(C)** Gender distribution and proportions of HA-MRSA-infected patients versus CA-MRSA carriers. **(D)** Distribution and proportions of clinical specimen types yielding HA-MRSA isolates.

To contextualize the burden of HA-MRSA in the clinical setting, we analyzed all clinical *S. aureus* isolates between January and December 2023: of 153 non-duplicate *S. aureus* strains (excluding repeated isolates from the same patient and same specimen type at different time points, while retaining those from the same patient but different specimen types; see Supplementary Table 2), 74 were confirmed as MRSA, resulting in an overall MRSA detection rate of 48.37% (74/153) among clinical *S. aureus* isolates. However, only 51 non-replicating MRSA strains were successfully revived and grew (detailed isolate information in Supplementary Table 3). Thus a total of 51 non-duplicate HA-MRSA isolates were used for further analysis. Key demographic and specimen-related characteristics of the 51 HA-MRSA isolates are as follows: in terms of age distribution, the isolates were obtained from patients aged 16–78 years, with no significant age skew (Figure 1A); for gender distribution, male patients accounted for a slightly higher proportion of HA-MRSA infections than females (52.94% vs. 47.06%), though the difference was minimal (Figure 1C); and regarding specimen source, wound secretions were the most common source (39.22%, 20/51), followed by lower respiratory tract specimens (including sputum and bronchoalveolar lavage fluid, 31.37%, 16/51), upper respiratory tract swabs (including nasal and throat swabs, 15.69%, 8/51), blood (5.88%, 3/51), sterile body fluids (5.88%, 3/51), and urine (1.96%, 1/51) (Figure 1D).

To assess MRSA nasal carriage in the community-residing Tibetan population (and individuals with recent community exposure), we collected 608 nasal cavity swabs from two groups: hospitalized patients within 24 h of admission (to exclude hospital-acquired colonization) and their family members. From these 608 swabs, 125 *S. aureus* isolates were identified; of these, 64 were confirmed as CA-MRSA, resulting in a CA-MRSA detection rate of 51.20% (64/125) among nasal *S. aureus* carriers (detailed isolate information in Supplementary Table 3). Key characteristics of the 64 CA-MRSA isolates are as follows: in terms of age distribution, the carriers of these isolates were aged 23–78 years (Figure 1B); and for gender distribution, females had a slightly higher CA-MRSA carriage rate than males (54.69% vs. 45.31%) (Figure 1C).

### Molecular typing characteristics of MRSA isolates

**FIGURE 2:**
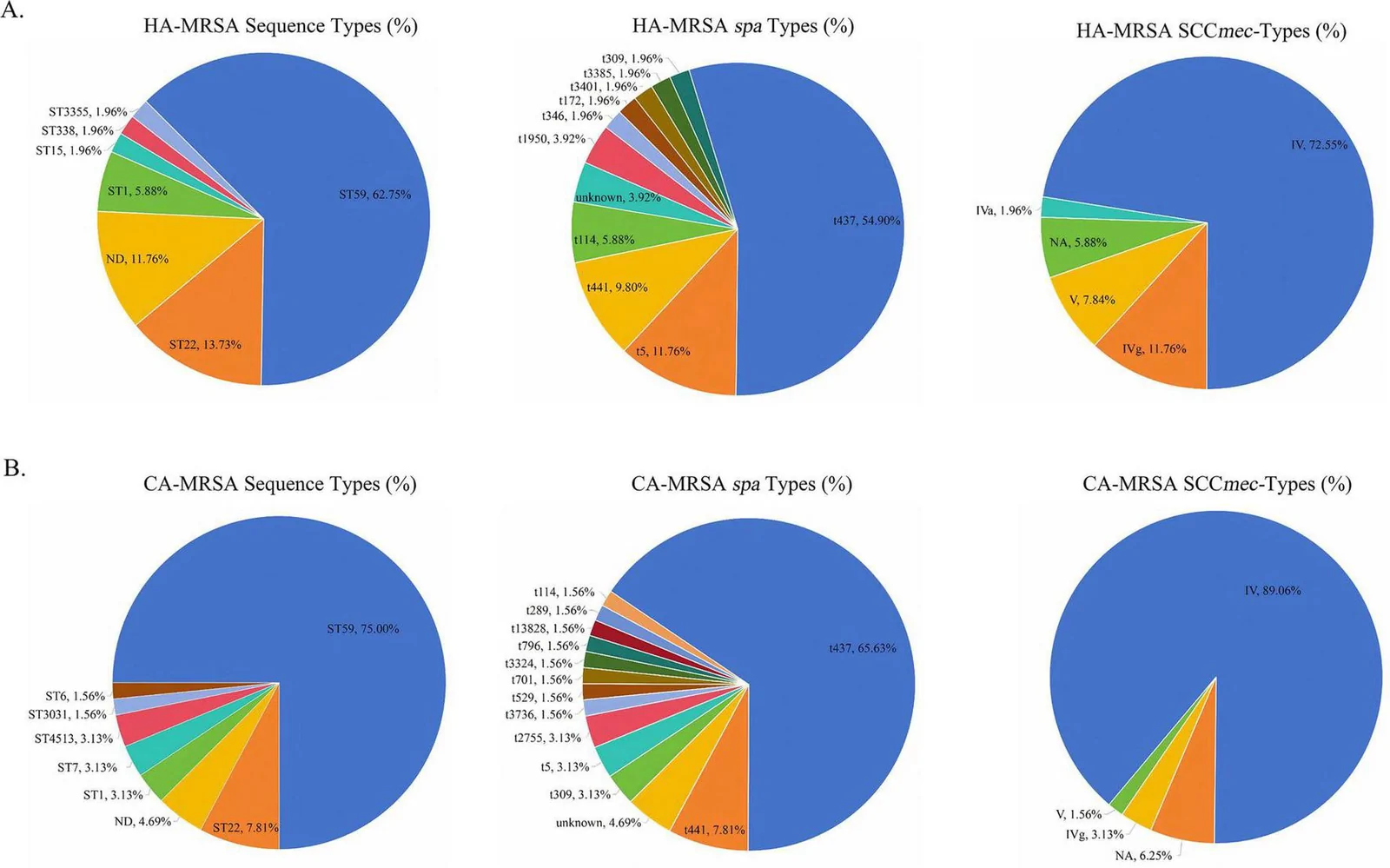
The proportions of different molecular types and molecular lineages among methicillin-resistant *Staphylococcus aureus* (MRSA) isolates. **(A)** Proportions of various STs, *spa* types, and SCC*mec* types among the 51 HA-MRSA isolates. **(B)** Proportions of various STs, *spa* types, and SCC*mec* types among the 64 CA-MRSA isolates.

To characterize the clonal diversity of MRSA in Tibet, we analyzed 51 HA-MRSA and 64 CA-MRSA isolates using four core molecular typing methods: clonal complex (CC), multilocus sequence typing (MLST), spa typing, and SCCmec typing. Among HA-MRSA isolates, we identified 6 distinct CCs (corresponding to 6 STs) and 10 spa types, while CA-MRSA isolates harbored 6 distinct CCs (7 STs) and 13 spa types. For CC profiles, CC59 was the dominant lineage in both subtypes: it accounted for 62.75% (32/51) of HA-MRSA (followed by CC22, 13.73% [7/51]; CC1, 5.88% [3/51]; and CC15/97/338, 1.96% each [1/51]) and 75% (48/64) of CA-MRSA (followed by CC22, 7.81% [5/64]; CC1, 3.13% [2/64]; CC7, 3.13% [2/64]; and CC6, 1.56% [1/64]) (Supplementary Table 3). For MLST profiles, ST59 was the dominant type in both subtypes: it accounted for 62.75% (32/51) of HA-MRSA (followed by ST22, 13.73% [7/51]; ST1, 5.88% [3/51]; and ST15/338/3355, 1.96% each [1/51]) and 75% (48/64) of CA-MRSA (followed by ST22, 7.81% [5/64]; ST1/7/4513, 3.13% each [2/64]; and ST6/3031, 1.56% each [1/64]) (Figures 2A, B). Furthermore, 11.76% (6/51) of HA-MRSA and 4.68% (3/64) of CA-MRSA isolates belonged to novel STs, indicating the existence of unique genetic lineages in the Tibetan MRSA population.

In *spa* typing, the 115 total isolates were discriminated into 18 unique *spa* types, with t437 being the most prevalent in both subtypes: it accounted for 54.90% (28/51) of HA-MRSA (followed by t5, 11.76% [6/51]) and 65.63% (42/64) of CA-MRSA (followed by t441, 7.82% [5/64]) (Figures 2A, B). A strong association between *spa* type and ST was observed: 88.57% (62/70) of t437 isolates belonged to the ST59 lineage, and 58.33% (7/12) of t5 isolates corresponded to ST22 (Supplementary Table 3), confirming the clonal linkage of these typing markers.

For SCC*mec* typing, four types (IV, IVa, IVg, V) were detected among the 115 isolates, with SCC*mec* IV being the predominant type in both HA-MRSA (72.55%, 37/51) and CA-MRSA (89.06%, 57/64) (Figures 2A, B); non-typable strains accounted for 6.09% (7/115) of the total.

Integrating all four typing methods, the most prevalent clone across the study was CC59-ST59-SCC*mec* IV-t437: it dominated CA-MRSA at 59.38% (38/64) and was the top clone in HA-MRSA at 39.22% (20/51). Other common clones included CC22-ST22-SCC*mec* IV-t5 (7.81%, 5/64 in CA-MRSA) and CC59-ST59-SCC*mec* IV-t441 (7.84%, 4/51 in HA-MRSA), further confirming the dominance of CC59-related lineages in both hospital and community settings.

### Phylogenetic analysis based on core genome SNPs

**FIGURE 3:**
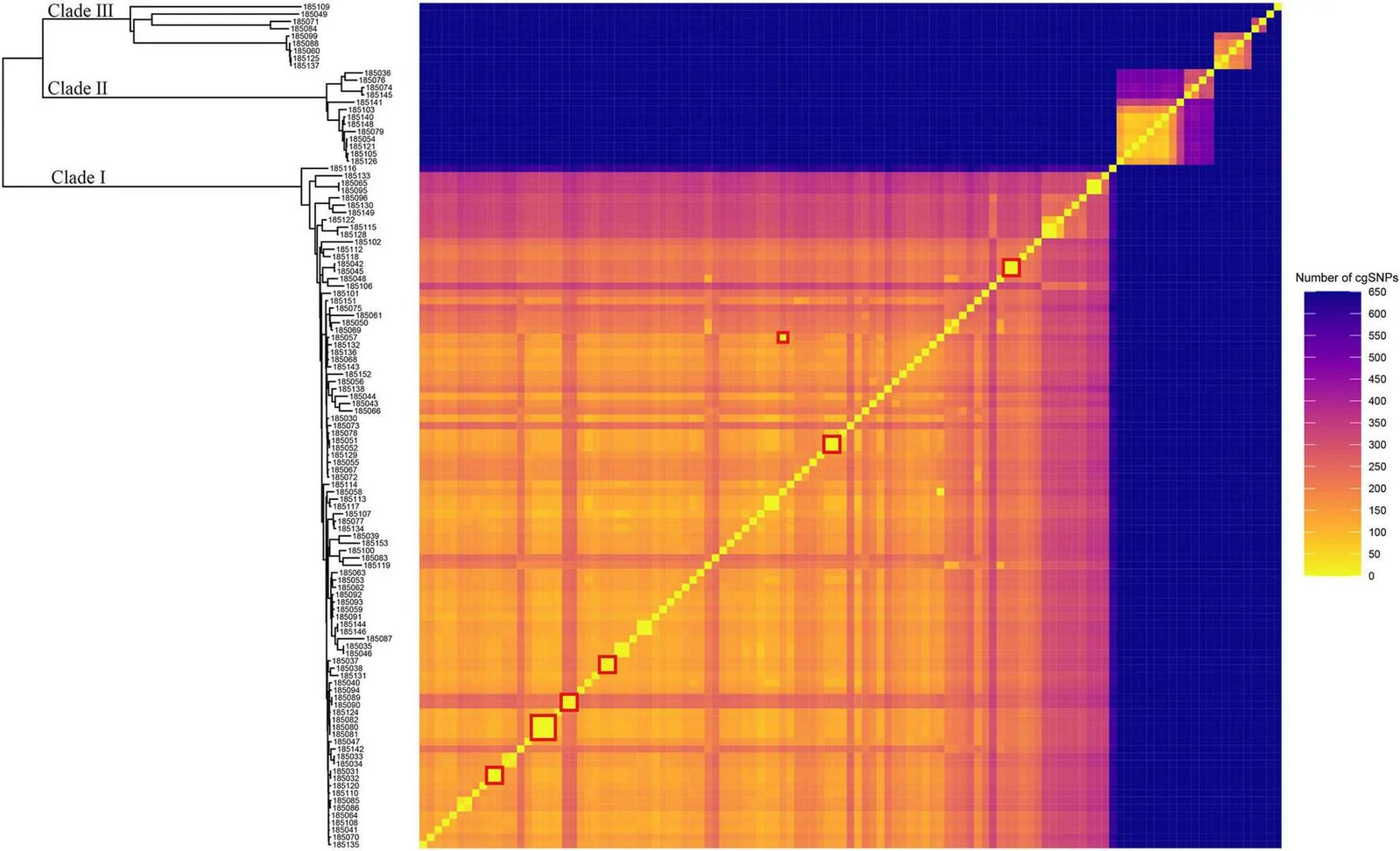
Phylogenetic reconstruction and core-genome SNP (cgSNP) analysis of 51 hospital-associated methicillin-resistant *Staphylococcus* aureus (HA-MRSA) and 64 community-colonizing methicillin-resistant *Staphylococcus aureus* (CA-MRSA) isolates. Maximum-likelihood tree inferred from cgSNPs (left) and a pairwise cgSNP distance heatmap (right), resolving three major clades (I–III). The red box highlights seven CA-MRSA transmission clusters defined by ≤25 cgSNP differences, approximating the ≤25 wgSNP threshold. Epidemiological links (shared household/hospital exposure) were confirmed within clusters.

To resolve the genetic heterogeneity and evolutionary relationships of Tibetan MRSA, we constructed phylogenetic trees using core-genome SNPs for comparative analysis. First, for the 115 MRSA isolates in this study (51 HA-MRSA, 64 CA-MRSA), phylogenetic clustering revealed three primary clades, each with distinct subclusters: all ST59 strains clustered exclusively within clade I, ST22 strains formed a monophyletic group in clade II, and the remaining non-ST59/ST22 isolates constituted clade III (Figure 3).

**FIGURE 4:**
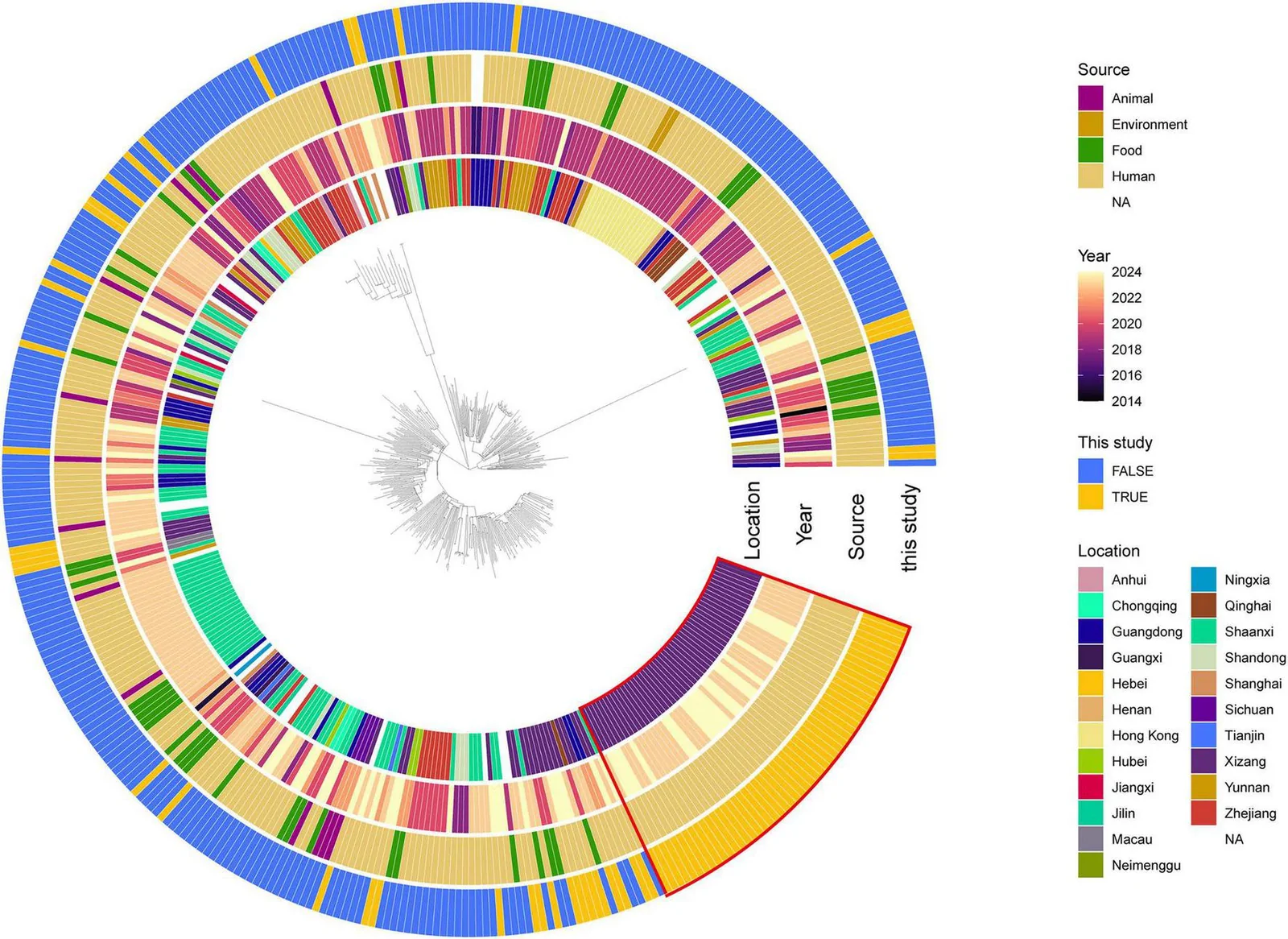
Phylogenetic structure of methicillin-resistant *Staphylococcus aureus* (MRSA) isolates from this study contextualized within a national collection of Chinese *S*. *aureus* genomes (2014–2024). Strain metadata, including geographic origin, isolation year, and source, are mapped on the tree (inner to outer circles).

To contextualize Tibetan MRSA within China’s national epidemic landscape, we retrieved 16,673 *S. aureus* genomes from the NCBI RefSeq database (accessed May 16, 2024), including 1,768 from China. After quality control, 1,678 high-quality genomes were used for downstream analysis. Among these, 288 were typed as ST59 (Supplementary Table 4); combining these with the 80 ST59 isolates from our study, we constructed a national ST59 *S. aureus* phylogenetic tree. This analysis revealed that the Tibetan ST59 isolates formed a relatively distinct phylogenetic cluster with limited genetic relatedness to strains from other Chinese provinces (highlighted by the red circle in Figure 4). Notably, despite screening this large national collection, no closely related intermediate lineages were identified, suggesting predominantly localized transmission within the Tibetan population.

We further investigated HA-MRSA and CA-MRSA transmission dynamics by quantifying pairwise cgSNP differences among the 115 isolates (after excluding duplicate isolates from the same patient, collected at different times or from different specimens). The total SNP distribution across all isolates ranged from 0 to 29,312 (Supplementary Table 5)-a broad range indicating significant genetic diversity, likely reflecting distinct evolutionary branches or geographical origins. Seven CA-MRSA transmission clusters were identified, defined by ≤25 cgSNP differences—a proxy for the widely accepted ≤25 wgSNP threshold for recent transmission (Coll et al., 2020). We identified seven CA-MRSA transmission clusters distinguished by ≤25 cgSNPs differences- a proxy for this wgSNP threshold. While most ST59 isolates exhibited >25 cgSNPs differences, consistent with limited recent epidemiological links, epidemiological records confirmed clear links within these clusters: isolates from the same household or individuals with documented spatiotemporal contact during hospitalization (Supplementary Table 3).

### Antimicrobial susceptibility testing and antimicrobial resistance gene analysis

To characterize the phenotypic resistance and genetic basis of antimicrobial resistance (AMR) in Tibetan MRSA, we conducted antimicrobial susceptibility testing (AST) on all 115 isolates (51 HA-MRSA, 64 CA-MRSA) and analyzed resistance gene profiles via WGS.

**FIGURE 5:**
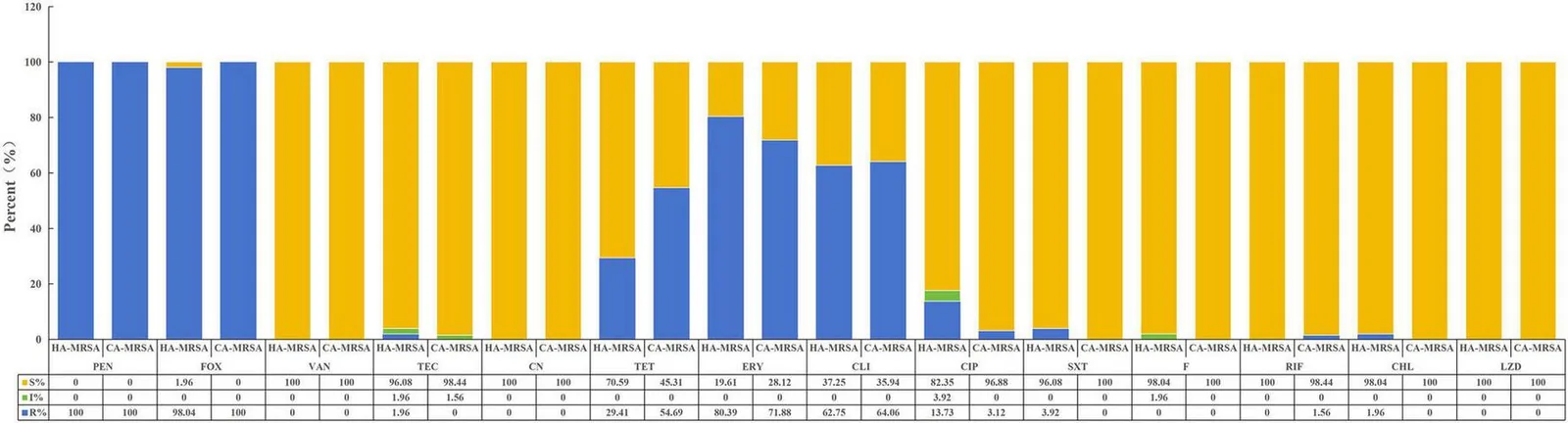
Antimicrobial susceptibility of hospital-associated methicillin-resistant *Staphylococcus* aureus (HA-MRSA) and community-colonizing methicillin-resistant *Staphylococcus aureus* (CA-MRSA) isolates. PEN, penicillin; FOX, cefoxitin; VAN, vancomycin; TEC, teicoplanin; CN, gentamicin; TET, tetracycline; ERY, erythromycin; CLI, clindamycin; CIP, ciprofloxacin; SXT, sulfamethoxazole/trimethoprim; F, nitrofurantoin; RIF, rifampin; CHL, chloramphenicol; LZD, linezolid. R%, proportion of resistant isolates; I%, proportion of intermediate isolates; S%, proportion of susceptible isolates.

AST results (Figure 5) revealed a consistent pattern of high resistance to β-lactam antibiotics across both subtypes, with 100% resistance to PEN in all isolates, and 100%/98.04% resistance to FOX in CA-MRSA/HA-MRSA isolates-reflecting the defining methicillin-resistant phenotype of MRSA. For non-β-lactam antibiotics, HA-MRSA and CA-MRSA showed comparable resistance rates, with minor differences: HA-MRSA exhibited resistance to ERY (80.39%, 41/51), CLI (62.75%, 32/51), and TET (29.41%, 15/51), while CA-MRSA had slightly higher resistance to ERY (71.88%, 46/64) and CLI (64.06%, 41/64) but lower resistance to TET (54.69%, 35/64). Low resistance rates (<15%) were observed for CIP (HA-MRSA 13.46%, CA-MRSA 3.12%), RIF (CA-MRSA 1.56%), SXT (HA-MRSA 3.92%), TEC (HA-MRSA 1.96%), and CHL (HA-MRSA 1.96%). Notably, all isolates were susceptible to clinically critical agents: VAN, CN, LZD, and F.

**FIGURE 6:**
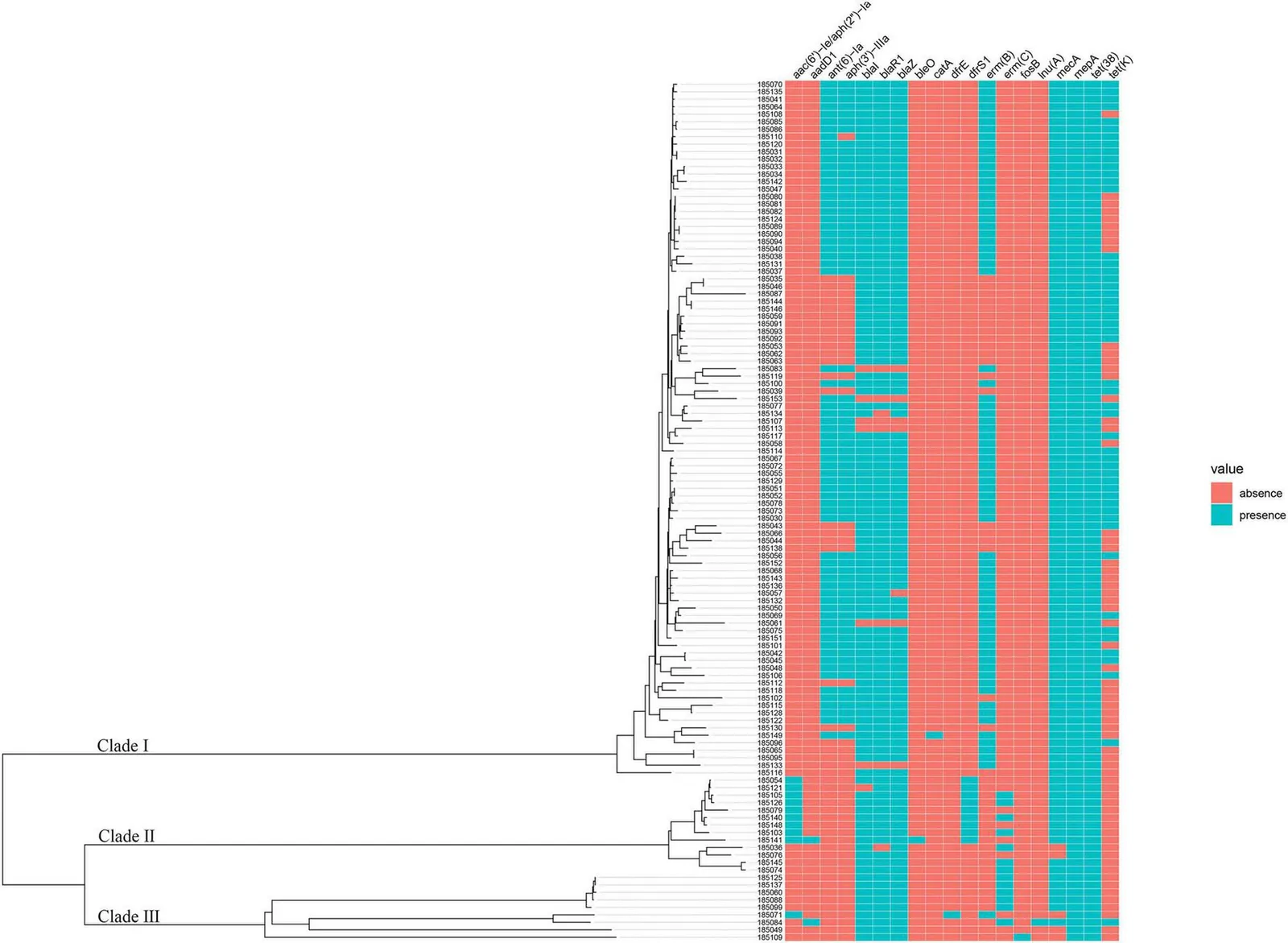
Phylogenetic clustering and antimicrobial resistance gene (ARG) heatmap of hospital-associated methicillin-resistant *Staphylococcus* aureus (HA-MRSA) and community-colonizing methicillin-resistant *Staphylococcus aureus* (CA-MRSA) isolates. Red blocks indicate absence; cyan blocks indicate presence.

WGS analysis identified 19 unique AMR genes across the 115 isolates, with distinct profiles between the major phylogenetic clades (Figure 6). Clade I (predominantly ST59) harbored core β-lactam resistance genes-*blaI, blaR1, blaZ* (mediating PEN resistance), and *mecA* (mediating methicillin/FOX resistance)-consistent with the PEN/FOX resistance phenotype. Most clade I isolates also carried *erm*(B) (conferring macrolide/lincosamide resistance, matching high ERY/CLI resistance rates), along with *tet(38)* and *tet(K)* (TET/tigecycline resistance) and aminoglycoside resistance genes *ant(6)-Ia* and *aph(3’)-IIIa*; one isolate additionally harbored *catA* (CHL resistance). In contrast, clade II (exclusively ST22) shared the core β-lactam resistance genes with clade I but differed in non-β-lactam resistance determinants: it used *aac(6’)-le/aph(2’)*-Ia for aminoglycoside resistance, *erm(C)* for macrolide/lincosamide resistance, and most isolates carried *dfr*S1 (trimethoprim resistance-consistent with rare SXT resistance in HA-MRSA).

Collectively, HA-MRSA and CA-MRSA exhibited similar overall resistance proportions across most antibiotics, a finding supported by overlapping AMR gene distributions between the two subtypes. Within the Tibetan MRSA population, resistance gene profiles varied primarily by ST rather than HA/CA origin: notably, ST59 isolates (the dominant lineage) displayed multidrug resistance to penicillins, cephalosporins, macrolides, lincosamides, and tetracyclines-aligning with the predominant resistance profile of ST59 MRSA reported in other regions of China.

### Distribution of virulence determinants

**FIGURE 7:**
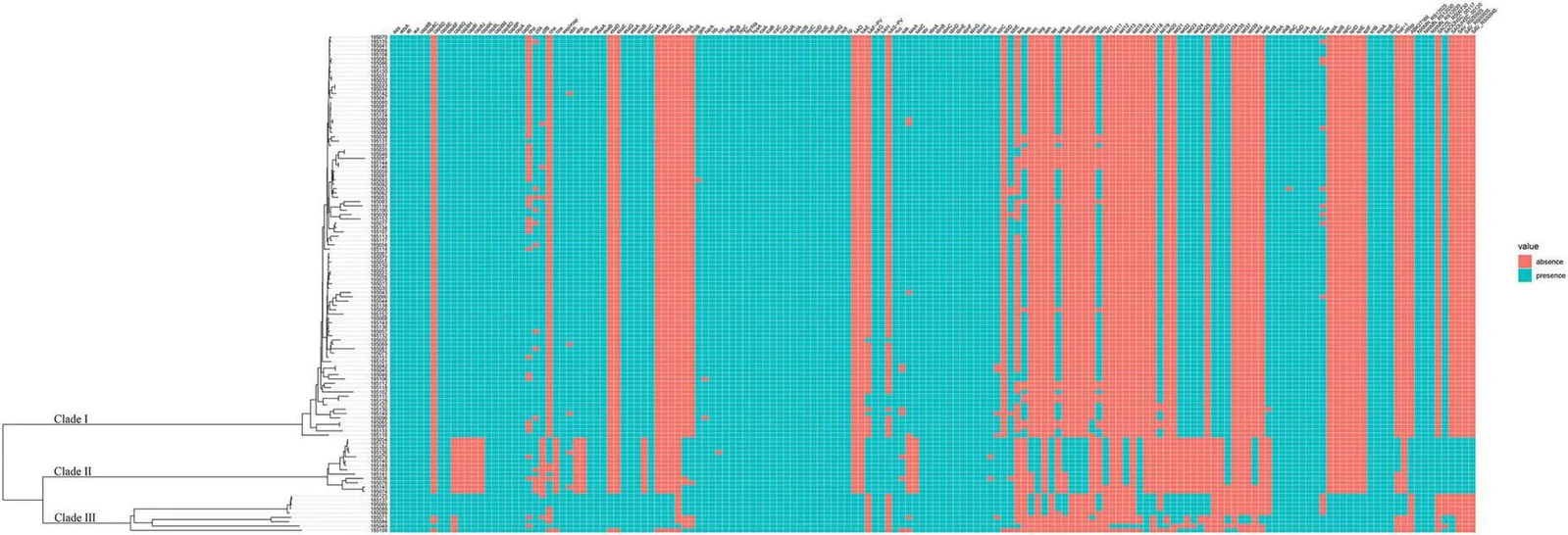
Phylogenetic clustering and virulence gene (VRG) heatmap of hospital-associated methicillin-resistant *Staphylococcus* aureus (HA-MRSA) and community-colonizing methicillin-resistant *Staphylococcus aureus* (CA-MRSA) isolates. Red blocks indicate absence; cyan blocks indicate presence.

To characterize the virulence potential of Tibetan MRSA and identify subtype-specific traits, we conducted a comparative genomic analysis of virulence genes across 115 isolates (51 HA-MRSA, 64 CA-MRSA). WGS identified 160 distinct virulence genes, of which 104 were highly conserved across most strains-representing the core virulence repertoire of Tibetan MRSA (Figure 7). These core genes were functionally grouped into six categories based on their role in pathogenesis: adhesion (*clfA/B, ebp, sasA/C, sdrC/E, sfaA/B/C/D*), invasion and toxins (*esaA/B/G, essA/B/C, esxA, hlb, hld, hly/hla, hlgA/B/C, lukG/H, seb, selK, selQ, set8/19/22/23/24/25/30/31/34*), immune evasion (*cap8B/C/E/F/G/H/I/J/K/L/M/O/P, capA/N, eap/map, efb, sbi, sak, spa*), iron uptake systems (*sbnA/B/C/D/E/F/G/H/I, sirA/B/C, isdA/B/C/D/E/F/G/I*), biofilm formation (*icaA/B/C/D/R, srtB*) and enzymes (*sspA/B/C*).

Virulence gene diversity was primarily driven by ST rather than HA/CA classification, with distinct profiles between the two major clades. For clade I (predominantly ST59), isolates exhibited 100% conservation of key virulence determinants including the capsule biosynthesis (*cap*8 cluster), iron acquisition systems (*sbn, sir, isd, sbn*), type VII secretory systems (*esaA/B/G, essA/B/C, esxA*), hemolysins (*hlgABC*, *hlb*, *hld, hly*/*hla*), bipartite leukocidin (*lukG/H*) and biofilm formation (*ica* locus). However, no significant differences of the virulence genes were observed between HA- and CA-MRSA strains in clade I (Figure 7). For clade II (exclusively ST22), isolates displayed a divergent virulence repertoire compared to clade I. ST22 lacked multiple key genes found in ST59, including the capsule synthesis operon (*cap8G/H/I/J/K*), adhesion genes (*clfb, ebp, efb, sasA/C*), coagulase (*coa*), the type VII secretion system component (*essC*), and the toxin genes (*set19/22/23/24/25*). In contrast, it uniquely encoded the adhesin (*sdrD*), the enterotoxin gene cluster *egc* (*seg*, *sei*, and *selM/N/O*), and toxins (*set1/4/13/16*) (Figure 7).

Panton-Valentine leukocidin (PVL)-a cytotoxin strongly linked to necrotizing skin and soft tissue infections (SSTIs) and mucosal lesions-exhibited a ST-specific distribution. In this study, PVL was detected in 18 isolates (15.65% of total), with detection observed across both HA-MRSA and CA-MRSA settings. Specimen sources were heterogeneous, encompassing nasal swabs, wound secretions/pus, and sputum, consistent with the broad ecological niche occupancy of the host strains. Critically, 100% of ST22 isolates (*n* = 12) were PVL-positive, whereas only 5 ST59 isolates (clade I, 6.25% of ST59) carried PVL. The remaining PVL-positive isolate belonged to a minor sequence type, reflecting sporadic acquisition of the *lukS-PV/lukF-PV*loci via phage transduction beyond the dominant clones (Figure 7). Furthermore, 14 isolates lacked the *spa*gene, which encodes immunoglobulin G-binding protein A-a critical immune evasion factor. This deficiency was distributed across both major STs: nine ST59 isolates (clade I) and five ST22 isolates (clade II). Given that *spa*mediates adherence to host extracellular matrix proteins and modulates innate immune responses, its loss may reduce immune recognition, thereby facilitating asymptomatic nasal carriage. Importantly, our CA-MRSA cohort comprised exclusively community-colonizing (nasal carriage) isolates rather than community-onset infection cases. This distinction likely explains the apparent inverse prevalence relative to studies of PVL in SSTIs, as we captured asymptomatic carriers rather than patients with active disease.

These findings reveal potential differences in virulence gene profiles between the two major lineages.ST59 (clade I) utilizes capsule-mediated immune evasion (*cap*8), multifactorial adhesion (*clfb*, *ebp*, *efb*, *sasA/C*), and coagulase activity (*coa*) to drive invasive colonization. Conversely, ST22 (clade II) employs *sdrD*-mediated adhesion, superantigen production (*egc*, *set1/4/13/16*), and enhanced leukocidin cytotoxicity (*lukS-PV/lukF-PV, lukG/H*), aligning with its association with toxin-mediated systemic diseases. However, as these observations are based solely on gene presence/absence analyses without functional validation (e.g., transcriptomic or phenotypic studies), the suggestion of distinct virulence strategies or altered gene expression as an explanation for the emergence of ST59 HA-MRSA remains speculative. Further experimental studies are warranted to confirm these hypotheses.

## Discussion

This study is the first to conduct genome-informed epidemiological monitoring of MRSA in Tibet since China established its national antimicrobial resistance surveillance network in 2009. Our key finding is an MRSA detection rate of 51.20% among *S*. *aureus*-colonized Tibetan individuals presenting to the study hospital, which exceeds the national average levels reported in recent CARSS surveillance data (China Antimicrobial Resistance Surveillance System, 2021, 2023, 2026). This notably high burden poses major challenges for prevention and control in Tibet. This elevated rate may be influenced by multiple factors, as suggested by previous studies in similar settings. Inadequate personal hygiene practices and lifestyle factors have been linked to higher bacterial transmission in rural and communal environments. The predominant availability and use of penicillin-class antibiotics in the region may exert selection pressure favoring resistant strains. Although penicillin exposure was not directly quantified in our cohort, this inference is consistent with procurement data from primary-care institutions in northwestern China (Gansu, 2012–2020) showing that penicillins (J01C) accounted for 45.15% of total antibiotic procurement (Cao et al., 2023). Close human-animal contact in agricultural and pastoral regions has been associated with cross-species MRSA transmission (Zou et al., 2022). The communal lifestyle of Tibetan populations may also contribute to transmission dynamics. However, these potential contributing factors were not directly investigated in the present study.

A second critical finding is the dominance of the CC59-ST59-SCC*mec* IV-t437 clone in both HA-MRSA (39.22% of isolates) and CA-MRSA (59.38% of isolates)-a pattern consistent with global and national trends. CC59 has emerged as a dominant MRSA lineage worldwide in recent decades and ST59 is also recognized as a predominant CA-MRSA clone in Asia (Chen and Huang, 2014). In China, ST59-MRSA has been identified as a leading clone in hospital and community settings across multiple provinces (Chen et al., 2021, 2022; Jin et al., 2021; Liu et al., 2016; Wang et al., 2022; Zhao et al., 2022), The success of the ST59-SCC*mec* IV-t437 subclone may be attributed to its enhanced biofilm-forming capacity, a phenotype reported for this subclone in Chinese pediatric isolates (Yang et al., 2017)-a trait that promotes persistent colonization on host tissues and medical devices, supporting both community transmission and hospital persistence. Notably, ST59 is also highly prevalent in the adjacent Sichuan Basin, particularly around Chengdu-a major transportation and tourism hub with frequent exchanges with Tibet. This geographic link suggests that increased tourism and population mobility between Tibet, Chengdu, and other Chinese regions may have facilitated the introduction and spread of ST59-MRSA in Tibet.

Our data reveal a distinct clonal structure of MRSA in Tibet compared with historical reports from other regions of China: ST59 predominated in our cohort, whereas ST239—historically a dominant HA-MRSA clone in China—was notably absent. While this observation is suggestive of a potential clonal shift, the possibility of low-frequency ST239 circulation cannot be excluded given our finite sample size. A plausible driver of this putative shift is the high prevalence of SCC*mec* IV (81.74% of our isolates)—the smaller, genetically streamlined cassette characteristic of community-associated clones compared with the bulkier SCC*mec* I–III elements typical of classical HA-MRSA. This reduced fitness cost likely facilitates the establishment and persistence of these clones in both community and hospital settings (D’Agata et al., 2009; Lakhundi and Zhang, 2018), enabling their emergence as successful lineages alongside—rather than necessarily through active displacement of—less adaptable HA-MRSA backgrounds. This dynamic, in which community reservoirs seed healthcare-associated infections, is increasingly recognized globally as a major driver of healthcare-associated infections (Otter and French, 2011).

The carriage of important virulence factor genes in *S. aureus* isolates of specific STs may be closely associated with their pathogenicity and disease progression. Chen et al. (2021) identified the chemotaxis inhibitory protein (Chp) as a key contributor to the heightened virulence of the ST59 lineage, which potentially drives MRSA lineage replacement in China. This heightened virulence is likely related to the high incidence of ST59 infections in hospitals. Furthermore, the prevalence of core genes from the immune evasion cluster (IEC), specifically *scn*, *chp*, and *sak*, may be key factors that lead to prolonged bacterial infection and progression to invasive infection, as previously reported in invasive *S*. *aureus* cohorts (Liu et al., 2025).

Methicillin-resistant *Staphylococcus aureus* lineages circulating in community carriage settings are often more virulent than typical hospital-associated clones (Deurenberg and Stobberingh, 2008), a difference partly driven by elevated expression of virulence-associated genes (Otto, 2013). A notable example is PVL, a cytotoxin closely linked to necrotizing skin and soft tissue infections (Lina et al., 1999). While PVL is common in community-associated MRSA in Europe and the United States (DeLeo et al., 2010), we observed the opposite pattern in Tibet: *lukS-PV/lukF-PV* prevalence was higher in HA-MRSA than in CA-MRSA isolates. Importantly, our CA-MRSA cohort comprised exclusively community-colonizing (nasal carriage) isolates rather than community-onset infection cases. This distinction likely explains the apparent inverse prevalence relative to studies of PVL in SSTIs, as we captured asymptomatic carriers rather than patients with active disease.

Globally, PVL prevalence among *S*. *aureus* varies widely—from <5% in healthcare-associated lineages to >30% in specific community-associated clones (Nurjadi et al., 2019; Zhao et al., 2012)—reflecting marked clonal and geographic variation. This ST-specific distribution aligns with prior reports identifying ST22 as a frequently PVL-positive lineage in China (Chen et al., 2014; Wang et al., 2016). The origin of community-associated PVL-positive MRSA remains unclear: such strains may arise via acquisition of the *mec* element by PVL-positive MSSA or, conversely, via phage-mediated transfer of *lukS-PV*/*lukF-PV*into methicillin-resistant backgrounds (Xiao et al., 2019).

Notably, our data reveal substantial clonal overlap of the dominant CC59-ST59-SCC*mec*IV-t437 clone between HA-MRSA and CA-MRSA (community colonization isolates), accompanied by highly similar antimicrobial resistance and virulence gene profiles. Although the seven transmission clusters defined by ≤25 cgSNP differences were observed exclusively among CA-MRSA isolates and no definitive HA-to-CA transmission events were demonstrated, these findings suggest that this successful clone circulates in both community and hospital settings.

Within the CA-MRSA cohort (i.e., community colonization isolates), seven such transmission clusters were identified, and epidemiological investigations revealed that isolates within these clusters shared either household or hospital exposure (Supplementary Table 3). Given that ST59 is a predominant MRSA clone circulating widely in East Asian communities - initially characterized among northern Taiwan children (Huang et al., 2008) and subsequently reported as dominant across diverse Asian settings (Chen and Huang, 2014; Chen et al., 2021) - these clusters may represent active transmission chains involving household or broader community exposure. We did not capture direct transmission events in this cross-sectional study, and some HA-MRSA isolates may represent pre-admission colonization despite the >48 h cutoff. Collectively, these data are consistent with the potential for circulation of the dominant clone in both settings, although causal transmission links remain unproven. Our findings support integrating whole-genome sequencing into regional surveillance to track how community reservoirs seed healthcare-associated infections—a pattern now evident across China (Chen and Huang, 2014) and in North American settings (David and Daum, 2010).

## Limitations

We acknowledge several limitations of this study. The cross-sectional design precludes definitive demonstration of transmission events; furthermore, some HA-MRSA isolates may represent pre-admission colonization despite the >48 h cutoff, and comparing colonization with clinical infection isolates may introduce epidemiological bias. Although only 51 of 74 HA-MRSA isolates were successfully revived, the sequenced subset retained representative demographic and clinical characteristics (Supplementary Table 3), suggesting that selection bias is unlikely to have substantially altered our major conclusions. Moreover, although phylogenetic inferences were derived strictly from core-genome alignments, interpreting ≤25 cgSNP differences as evidence of recent transmission remains subject to inherent phylogenetic uncertainty. Finally, uneven geographic sampling of comparator genomes and the lack of direct data on environmental and behavioral risk factors may limit interpretations of the elevated MRSA burden in Tibet.

## Conclusion

Overall, the CC59-ST59-SCC*mec* IV-t437 clone constitutes the predominant MRSA lineage in Tibet, forming a distinct phylogenetic cluster. Extensive clonal overlap between HA-MRSA and CA-MRSA isolates, coupled with the identification of PVL-positive ST22 strains, defines a complex epidemiological setting in which community reservoirs likely seed healthcare-associated infections. These findings support the integration of WGS into regional surveillance to track high-risk clones and inform targeted infection control strategies tailored to this high-burden population.

## Data Availability

The datasets presented in this study can be found in online repositories. The names of the repository/repositories and accession number(s) can be found in the article/Supplementary material.

## Footnotes

1
https://pubmlst.org/organisms/staphylococcus-aureus

2
https://cge.food.dtu.dk/services/spaTyper/

3
https://cge.food.dtu.dk/services/SCCmecFinder/

4
https://genepi.dk/virulencefinder

5
http://www.mgc.ac.cn/VFs/main.htm

## Supplementary material

Supplementary Table 1 Detailed assembly statistics for each isolate, including sequencing depth, N50, number of contigs, and total assembly length and GC content.

Supplementary Table 2 Description of 153 *S. aureus* strains clinical data from January to December 2023.

Supplementary Table 3 Detailed characteristics of the studied methicillin-resistant *Staphylococcus aureus* (MRSA) isolates, including source, molecular type, and epidemiological data for 51 hospital-associated methicillin-resistant *Staphylococcus* aureus (HA-MRSA) clinical isolates and 64 community-colonizing methicillin-resistant *Staphylococcus aureus* (CA-MRSA) carriage isolates.

Supplementary Table 4 Description of 288 ST59 *S. aureus* strains from China. Data were retrieved from NCBI.

Supplementary Table 5 Pairwise single nucleotide polymorphism (SNP) distances between methicillin-resistant *Staphylococcus aureus* (MRSA) isolates.
